# Development of a Biomarker Panel to Distinguish Risk of Progressive Chronic Kidney Disease

**DOI:** 10.3390/biomedicines8120606

**Published:** 2020-12-14

**Authors:** Evan Owens, Ken-Soon Tan, Robert Ellis, Sharon Del Vecchio, Tyrone Humphries, Erica Lennan, David Vesey, Helen Healy, Wendy Hoy, Glenda Gobe

**Affiliations:** 1 NHMRC CKD CRE (CKD.QLD), The University of Queensland, Brisbane 4067, Australia; evan.owens@uq.edu.au (E.O.); Ken-Soon.Tan@health.qld.gov.au (K.-S.T.); Helen.Healy@health.qld.gov.au (H.H.); 2 Faculty of Medicine, The University of Queensland, Brisbane 4067, Australia; r.ellis1@uq.edu.au (R.E.); sharon.delvecchio@uq.net.au (S.D.V.); tyrone.humphries@uq.net.au (T.H.); david.vesey@health.qld.gov.au (D.V.); 3 Kidney Disease Research Collaborative, Translational Research Institute, Princess Alexandra Hospital, The University of Queensland, Brisbane 4102, Australia; 4 Renal Medicine, Metro South Hospital and Health Service, Logan Hospital, Meadowbrook 4131, Australia; Erica.Lennan@health.qld.gov.au; 5 Kidney Health Service, Royal Brisbane and Women’s Hospital, Brisbane 4029, Australia; 6 Centre for Chronic Disease, Faculty of Medicine, The University of Queensland, Brisbane 4067, Australia

**Keywords:** chronic kidney disease, progression, biomarkers, progressive

## Abstract

Chronic kidney disease (CKD) patients typically progress to kidney failure, but the rate of progression differs per patient or may not occur at all. Current CKD screening methods are sub-optimal at predicting progressive kidney function decline. This investigation develops a model for predicting progressive CKD based on a panel of biomarkers representing the pathophysiological processes of CKD, kidney function, and common CKD comorbidities. Two patient cohorts are utilised: The CKD Queensland Registry (n = 418), termed the Biomarker Discovery cohort; and the CKD Biobank (n = 62), termed the Predictive Model cohort. Progression status is assigned with a composite outcome of a ≥30% decline in eGFR from baseline, initiation of dialysis, or kidney transplantation. Baseline biomarker measurements are compared between progressive and non-progressive patients via logistic regression. In the Biomarker Discovery cohort, 13 biomarkers differed significantly between progressive and non-progressive patients, while 10 differed in the Predictive Model cohort. From this, a predictive model, based on a biomarker panel of serum creatinine, osteopontin, tryptase, urea, and eGFR, was calculated via linear discriminant analysis. This model has an accuracy of 84.3% when predicting future progressive CKD at baseline, greater than eGFR (66.1%), sCr (67.7%), albuminuria (53.2%), or albumin-creatinine ratio (53.2%).

## 1. Introduction

Chronic kidney disease (CKD) is a major health and economic burden worldwide, including Australia [1,2]. Irrespective of aetiology, many patients progress towards kidney failure requiring dialysis or kidney transplantation [3]. Currently, there are no clinically robust biomarkers to predict progressive CKD. Rather, clinicians rely on multiple longitudinal kidney measurements, such as estimated glomerular filtration rate (eGFR), albuminuria (Alb) and albumin-creatinine ratio (ACR), to identify progression [4]. However, these traditional biomarkers have the limited predictive capacity for progressive CKD [5].

Although lower eGFR and elevated ACR are associated with an increased risk of kidney failure, these biomarkers do not adequately predict worse clinical outcomes in patients with minimal kidney damage or reduced kidney function [6]. Additionally, CKD patients can display one of several non-linear eGFR trajectories, including stable or increasing eGFR, thereby reducing the predictive utility of longitudinal kidney measurements [7,8]. Thus, it can be argued that if a subset of CKD patients is unlikely to experience progression, an undifferentiated approach to clinical management is an inefficient use of health resources and introduces the risk of over-treatment. There have been several attempts to create a model for predicting worse clinical outcomes, such as kidney failure and progressive CKD, with the most notable and robust being The Kidney Failure Risk Equation [9]. However, these tools remain inaccurate because of various limitations.

This investigation aimed to develop predictive models, termed the Distinguishing Risk of Progressive (DROP) CKD models, for predicting progressive CKD. It was hypothesised that a panel of biomarkers representing the pathophysiological processes underpinning progressive CKD, traditional kidney measurements, and common CKD comorbidities would be more accurate in predicting progression than traditional kidney measurements currently used. Compared to eGFR, sCr, Alb or ACR, the DROP CKD models were more accurate at predicting progressive CKD.

## 2. Material and Methods

An investigative study was conducted to determine the association between baseline measurements of blood and urinary biomarkers and progressive CKD. In addition, a predictive model for progressive CKD was constructed based on a panel of these biomarkers. This study included a biomarker discovery-based component using one cohort, the CKD Queensland (CKD QLD) Registry cohort and a predictive model development component in the other cohort, the CKD Biobank cohort. Biomarkers that were associated with progressive CKD in the CKD QLD cohort, in addition to biomarkers identified in the literature, were included in the predictive model development with the CKD Biobank cohort.

### 2.1. Study Population

A sample of 418 patients was ascertained from the CKD QLD Registry (termed the Biomarker Discovery cohort), a registry of patients who are known to specialist nephrology practices across Queensland, Australia, with pre-terminal CKD and with records of associated clinical data. These patients were recruited via an opt-in consent model [10] (https://cre-ckd.centre.uq.edu.au/CKD.QLD). Patients were included if ≥4 independent eGFR measurements were recorded over a minimum of 12 months during follow-up. This subset of patients was recruited from the Royal Brisbane and Women’s Hospital (RBWH) between May-2011 and May-2015 and they were followed until the date of kidney replacement therapy (dialysis/transplant), death, discharge, loss to follow-up, or censor date of 30 June 2017.

A sample of 62 patients was ascertained from the CKD Biobank (termed the Predictive Model cohort), a repository of pre-terminal CKD patients, with associated clinical data and baseline biospecimens, who are known to specialist nephrology practices across Queensland, Australia and are recruited via a broad consent model [11] (https://cre-ckd.centre.uq.edu.au/project/nhmrc-ckd-biobank). Patients were included if ≥2 independent eGFR measurements were recorded during follow-up. This subset of patients was recruited from the Logan Hospital, Queensland, between November 2017 and October 2018, and was followed until kidney replacement therapy or censor date of 31 December 2019.

This study was approved by The University of Queensland (UQ) Human Research Ethics Committee (HREC) (Approval Number: 2016001395) in October 2016. The CKD QLD Registry was approved by UQ HREC (Approval Number: 2011000029) in January 2011 and the Queensland Health Ethics Office (Approval Number: HREC/10/QHC/41) in November 2011. The latter approval was superseded by the RBWH HREC (Approval Number: HREC/15/QRBW/294) in May 2015. The CKD Biobank was approved by the RBWH HREC (HREC/15/QRBW/610) in March 2016. This study was carried out in accordance with the National Statement on Ethical Conduct in Human Research (2007) produced by the National Health and Medical Research Council of Australia. This statement has been developed with reference to the Declaration of Helsinki to protect the interests of people who agree to participate in human research studies.

### 2.2. Outcome

The outcome for both cohorts was subsequent progressive CKD occurring during follow-up. This was defined by a composite outcome of a ≥30% decline in eGFR from baseline, initiation of dialysis, or kidney transplantation. A ≥30% decline in eGFR from baseline was chosen to represent a progressive decline kidney function because the CKD Prognosis Consortium found it conferred a substantial risk of kidney failure in CKD with an eGFR 60+ or <60 mL/min/1.73 m^2^ [12].

### 2.3. Biomarkers

A panel of 61 biomarkers (Table S1) was assessed in the Biomarker Discovery cohort. Laboratory data, including routine kidney function measurements from Queensland Health Pathology Services (QHPS) and taken during the clinical management of CKD patients, were sourced from Queensland Health integrated electronic Medical Record (ieMR) or other databases. A biomarker was included for analysis if it was measured at baseline eGFR measurement or ≤3 months prior to the baseline eGFR measurement in an individual patient and was measured in ≥50 patients. In the Biomarker Discovery cohort, eGFR was calculated by the 2009 CKD-EPI creatinine equation.

A panel of 37 biomarkers was assessed in the Predictive Model cohort. The kidney measurements sCr, Alb, eGFR (calculated by the 2012 CKD-EPI creatinine-cystatin C equation), and ACR were measured in baseline serum and urine biospecimens by QHPS. Fetuin-B (RD191172200R; BioVendor; Brno, Czech Republic) and the mast cell proteases tryptase, chymase, and carboxypeptidase A3 (abx153400, abx151070, abx151163; Abbexa; Cambridge, UK) were measured in baseline serum biospecimens by ELISA. Cystatin-C, kidney injury molecule-1 (KIM-1), neutrophil gelatinase-associated lipocalin, tissue factor (TF), stem cell factor (SCF), colony-stimulating factor-2, monocyte chemoattractant protein-1, interleukin-1β, interleukin-6, chemokine ligand 5, osteopontin, tumour necrosis factor (TNF)-α, soluble TNF receptor (sTNFR)-1, sTNFR-II, hepatocyte growth factor, basic fibroblast growth factor, collagen IV α1, D-Dimer and fibrin degradation products, and matrix metallopeptidase-1 and matrix metallopeptidase-9 were measured in baseline plasma biospecimens by custom multiplex Luminex assays (R&D Systems; Minneapolis, United States of America). Protein carbonyl content was measured in baseline plasma biospecimens via 2,4-dinitrophenylhydrazine staining [13]. Uromodulin (RD191163200R; BioVendor, Brno, Czech Republic) was measured in baseline urine biospecimens by ELISA. Calcium, bicarbonate, phosphate, chloride, and urea measured in the serum and haematocrit and haemoglobin measured in whole blood were sourced from QHPS data and from ieMR and other databases. These biomarker measurements were measured at baseline eGFR or ≤3 months prior to the baseline eGFR measurement in an individual patient.

### 2.4. Statistical Analysis

Patient characteristics of progressive and non-progressive CKD patients were compared using an independent *t*-test or Mann-Whitney U-test, depending on whether continuous variables were distributed normally. A Chi-Square test of homogeneity was used to compare categorical variables. If a characteristic was found to differ between progressive and non-progressive patients, the relative risk was calculated.

Biomarker concentrations were compared between progressive and non-progressive CKD patients using logistic regression. For each biomarker, the basic covariates of age, gender, kidney disease diagnosis, body mass index, and follow up time were included in the logistic regression. Regarding an independent biomarker—if a covariate did not significantly contribute to the model, it was dropped from the logistic regression.

Several predictive models for predicting progressive CKD, termed the DROP CKD models, based on biomarker expression were calculated via linear discriminant analysis. This is a statistical approach for predicting class membership of individuals. Biomarkers that were observed as differing between progressive and non-progressive CKD patients of the Predictive Model cohort were selected for inclusion in the development of these predictive models. Biomarker selection was via a step approach, which included (step-forward) or excluded (step-backward) a biomarker if it improved the accuracy of the model. If the inclusion or exclusion of a biomarker did not improve the accuracy of the predictive model, the selection process was stopped, and the biomarker panel of the previous step was confirmed as the final predictive model.

Predictive models were calculated with and without the basic covariates of age, gender, kidney disease diagnosis, body mass index, and follow-up time included using both step-forward and step-backwards approaches. Additionally, predictive models were calculated for the routine kidney measurements sCr, eGFR, Alb, and ACR. The accuracy of these predictive models, in terms of predicting future progression status (based on baseline biomarker expression), were compared with each other and to the predictive models of the routine kidney measurements.

Statistical analysis was performed using R (Version 3.3.1) with the packages “MASS” and “sm”. The specific code for these packages is available online [14]. Statistical significance was assigned at *p* < 0.05.

## 3. Results

### 3.1. Patient Characteristics at Baseline

Patients were retrospectively classified as progressive or non-progressive based on a composite outcome of a ≥30% decline in eGFR from baseline, initiation of dialysis, or kidney transplantation. Patients of the Biomarker Discovery cohort (n = 418) that were classified as progressive (n = 183) only differed from those classified as non-progressive (n = 235) in follow-up time (Table 1). Progressive patients were followed for a significantly longer time (*p* < 0.0001), 4.6 ± 1.5 years, compared to non-progressive patients who were followed for 3.9 ± 1.6 years. A longer follow-up time, ≥4.3 years (the median follow-up time), conferred a relative risk of 1.3 95% CI [1.1, 1.7] for progression compared to a shorter follow-up time ≤ 4.3 years.

In the Predictive Model cohort (n = 62), patients retrospectively classified as progressive (n = 33) and non-progressive (n = 29) only differed in the eGFR category distribution (Table 1). Progressive patients were skewed towards more advanced eGFR categories, while non-progressive patients were skewed towards less advanced eGFR categories (χ^2^(4, 68) = 10.1, *p* < 0.05). A more advanced eGFR category, with a baseline eGFR ≤ 60 mL/min/1.73 m^2^, conferred a relative risk 1.7 95% CI [1.0, 3.1] for progression compared to less advanced eGFR categories with a baseline eGFR > 60 mL/min/1.73 m^2^.

### 3.2. Assessing the Composite Outcome

**Figure 1:**
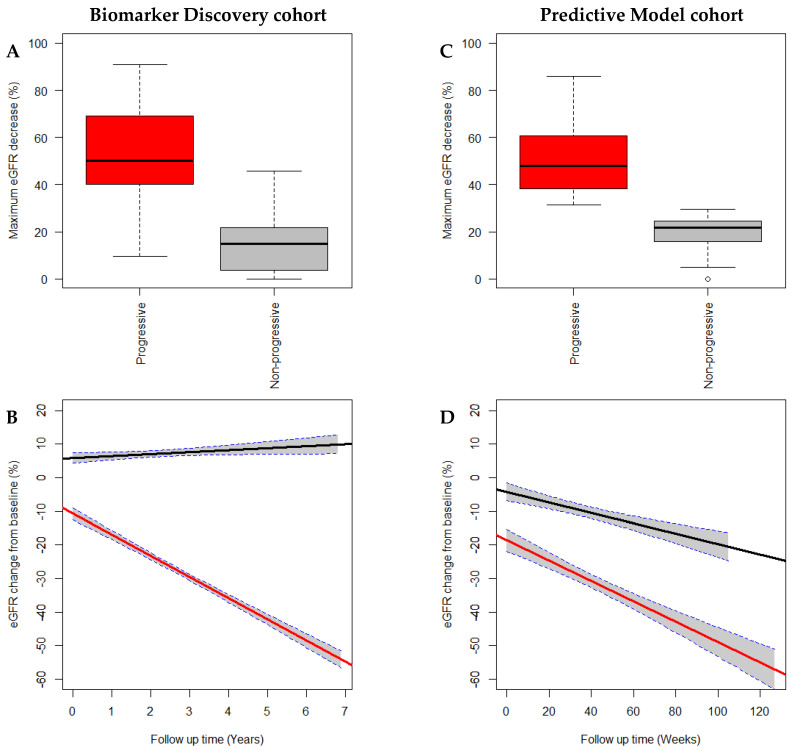
CKD patients of the Biomarker Discovery (**A**,**B**) and the Predictive Model (**C**,**D**) cohorts were classified as progressive (red) or non-progressive (grey/black) based on a composite outcome of a ≥30% decline in eGFR from baseline, initiation of dialysis, or kidney transplantation. This definition distinctly classified CKD patients as progressive and non-progressive. Progressive patients demonstrated a larger maximum eGFR decrease (%) from baseline compared to non-progressive patients (**A**,**C**). Progressive patients of the Biomarker Discovery cohort demonstrated a negative trajectory for eGFR percentage change from the baseline, which differed significantly (*p* < 0.0001) from non-progressive patients who demonstrated a positive trajectory (**C**). Both progressive and non-progressive patients of the Predictive Model cohort demonstrated negative trajectories; however, these were significantly different from each other with progressive patients demonstrating a more negative trajectory (**D**). Abbreviations: Chronic kidney disease (CKD), estimated glomerular filtration rate (eGFR).

The composite outcome was found to distinguish between progressive and non-progressive CKD patients by the maximum eGFR percentage decrease from baseline and the longitudinal trajectory of eGFR percentage change from baseline (Figure 1). Progressive patients of the Biomarker Discovery cohort experienced a greater maximum eGFR percentage decrease of 54.4 ± 13.8% compared to 17.6 ± 10.2% for non-progressive patients (*p* < 0.0001). This was also observed in the Predictive Model cohort with progressive patients experiencing a maximum eGFR percentage decrease of 50.9 ± 14.8% compared to 20.1 ± 7.4% for non-progressive patients (*p* < 0.0001).

Both progressive and non-progressive patients of the Biomarker Discovery cohort demonstrated significantly different longitudinal trajectories of eGFR percentage change from baseline (F(3, 6609) = 961.8, *p* < 0.0001) with progressive patients experiencing a steep decline and non-progressive patients experiencing a shallow increase in the percentage change in eGFR from baseline. Within the Predictive Model cohort, both patient groups demonstrated longitudinal downwards trajectories; however, these trajectories were significantly different (F(3, 454) = 98.87, *p* < 0.01). Progressive patients experienced a steep decline, while non-progressive patients experienced a shallower decline in the percentage change in eGFR from baseline.

### 3.3. Biomarker Discovery Cohort—Baseline Biomarker Concentrations

Of the 61 biomarkers screened in the CKD QLD cohort (Table S1), 13 differed significantly between patients classified as progressive and non-progressive at baseline (Table 2), while 48 biomarkers did not differ between patient groups (Table S2A,B). The kidney measurements sCr (*p* < 0.01), urea (*p* < 0.001), and protein creatinine ratio (*p* < 0.001) were increased in progressive patients by 20.3 ± 6.1 µmol/L, 2.4 ± 0.6 mmol/L, and 25.4 ± 19.6 mg/L, respectively, while eGFR (*p* < 0.05) was decreased in progressive patients by 3.3 ± 1.4 mL/min/1.7 m^2^. Bicarbonate (*p* < 0.001) was decreased by 1.1 ± 0.3 mmol/L in progressive patients, while chloride (*p* < 0.001) was increased by 1.0 ± 0.4 mmol/L. Haematocrit (*p* < 0.0001) and haemoglobin (*p* < 0.0001) were decreased by 0.02 ± 0.01 and 8.8 ± 1.8 g/L in progressive patients. Parathyroid hormone (*p* < 0.01), phosphate (*p* < 0.01), and alkaline phosphatase (*p* < 0.05) were increased in progressive patients by 49.9 ± 17.8 ng/L, 0.1 ± 0.0 mmol/L, and 6.7 ± 4.4 U/L, respectively, while calcium (*p* < 0.01) was decreased by 0.03 ± 0.01 mmol/L in progressive patients. Ferritin (*p* < 0.05) was increased by 68.4 ± 28.5 µg/L in progressive CKD patients.

### 3.4. Predictive Model Cohort—Baseline Biomarker Concentrations

Of the 37 biomarkers screened, 10 differed significantly between progressive and non-progressive CKD patients at baseline (Table 2), while 23 biomarkers did not (Table S3A,B). sCr (*p* < 0.001) and urea (*p* < 0.01) were increased in progressive patients by 98.9 ± 23.4 µmol/L and 4.5 ± 1.6 mmol/L, respectively. Additionally, eGFR was decreased by 28.5 ± 9.0 mL/min/1.73 m^2^ in progressive patients (*p* < 0.01). The tissue injury biomarkers osteopontin (*p* < 0.01), and TF (*p* < 0.001) were increased by 10.7 ± 6.1 ng/mL and 23.1 ± 7.6 ng/mL, respectively. Several inflammatory biomarkers differed significantly between progressive and non-progressive CKD patients. The TNF-α receptors sTNFR-I (*p* < 0.01) and sTNFR-II (*p* < 0.05) were increased by 1.1 ± 0.4 ng/mL and 1.5 ± 0.9 ng/mL, respectively. The mast cell biomarkers SCF (*p* < 0.001) and tryptase (*p* < 0.01) were increased by 67.7 ± 17.2 pg/mL and 1.8 ± 0.8 ng/mL in progressive patients. Bicarbonate was decreased in progressive CKD patients by 2.3 ± 0.9 mmol/L (*p* < 0.01).

### 3.5. DROP CKD—A Predictive Model

**Figure 2:**
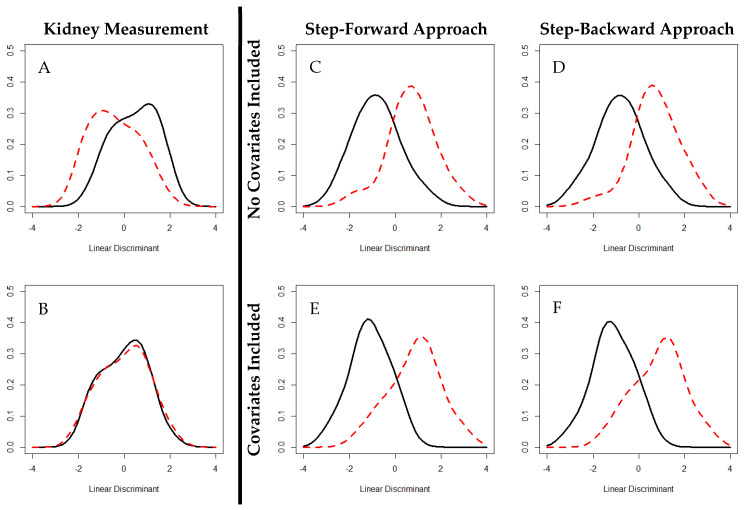
Linear discriminants of kidney measurements and DROP CKD models. Distinguishing Risk of Progressive CKD models, calculated via linear discriminant analysis, were more accurate than those calculated for the kidney measurements eGFR (**A**) and albuminuria (**B**) when predicting future progressive CKD at baseline in the Predictive Model cohort. eGFR and albuminuria conferred accuracies of 66.1 % and 53.2%, respectively. The step-forward (**C**) approach calculated a predictive model with an accuracy of 84.3% with the biomarkers sCr, eGFR, osteopontin, tryptase, and urea. When including basic covariates (age, body mass index, follow-up time, kidney disease diagnosis, and gender) the step-forward approach (**D**) had an accuracy of 83.3%. The step-backward approach (**E**) calculated a predictive model with an accuracy of 86.3% with the bicarbonate biomarkers, osteopontin, SCF, tissue factor, tryptase, urea, sCr, and eGFR. When including basic covariates, the step-backward approach (**F**) had an accuracy of 81.25% Frequency of linear discriminants was plotted by progressive (dashed, red) and non-progressive (solid, black) CKD. Greater separation of progressive and non-progressive distributions indicates greater accuracy when predicting progressive CKD by a predictive model. Abbreviations: Estimated glomerular filtration rate (eGFR), serum creatinine (sCR), stem cell factor (SCF).

Created either through a step-forward or step-backward approach, the DROP CKD models were more accurate at predicting progressive CKD based on baseline biomarker expression in the Predictive Model cohort than the traditional kidney function measurements sCr, eGFR, Alb or ACR in solitude (Figure 2). The step-forward approach selected a biomarker panel of sCr, osteopontin, tryptase, urea, and eGFR and had an accuracy of 84.3% and 83.3%, including the basic covariates age, body mass index, follow-up time, kidney disease diagnosis, and gender. The step-backward approach selected a biomarker panel of bicarbonate, osteopontin, SCF, tissue factor, tryptase, urea, sCr, and eGFR with an accuracy of 86.3%. When including the basic covariates, the accuracy of the model created via the step-backward approach decreased to 81.3%. These were in comparison to the kidney measurements sCr, eGFR, Alb, and ACR which had an in predictive solitude accuracy of 67.7%, 66.1%, 53.2%, and 53.2%, respectively, and a cumulative predictive accuracy of 75.8%.

## 4. Discussion

**Figure 3:**
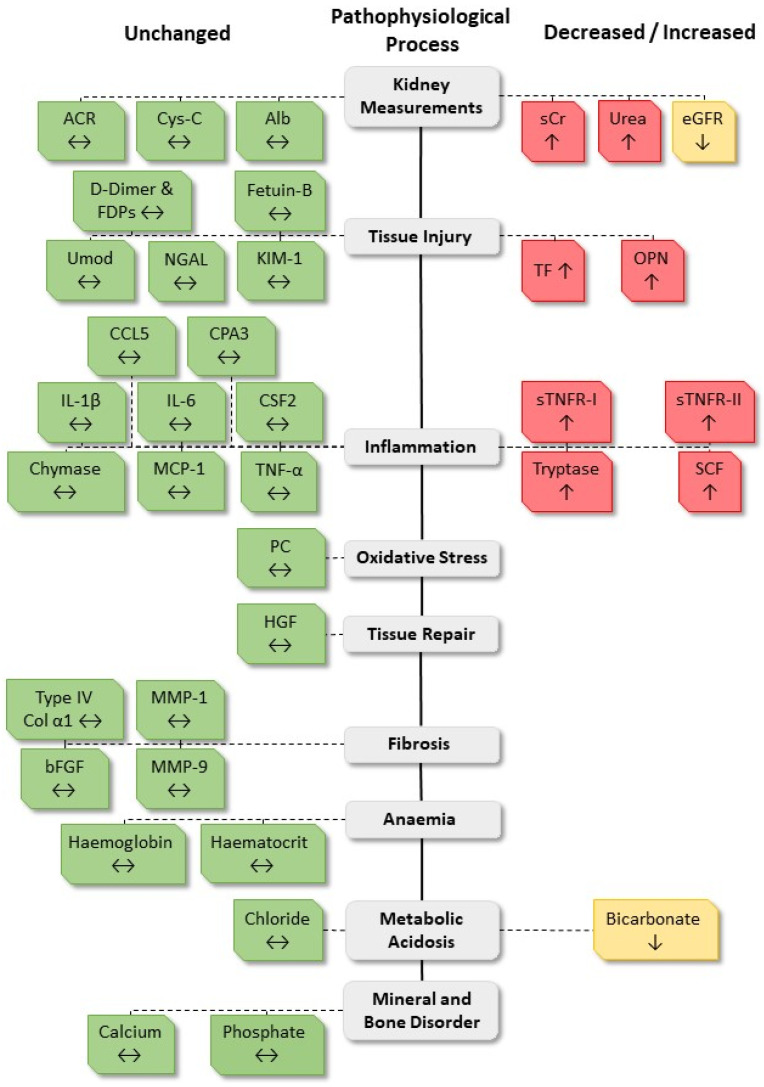
Biomarker expression that differed between progressive and non-progressive patients of the Predictive Model cohort. A schematic diagram of the 37 biomarkers screened in the Predictive Model cohort and whether the concentration was increased (red), decreased, or unchanged (green) between progressive and non-progressive patients. Abbreviations: Albumin-creatinine ratio (ACR), cystatin-c (Cys-C), albuminuria (Alb), serum creatinine (sCr), estimated glomerular filtration rate (eGFR), fibrin degradation products (FDPs), uromodulin (Umod), neutrophil gelatinase-associated lipocalin (NGAL), kidney injury molecule-1 (KIM-1), chemokine ligand 5 (CCL5), carboxypeptidase A3 (CPA3), interleukin (IL), colony-stimulating factor 2 (CSF2), monocyte chemoattractant protein-1 (MCP-1), tumour necrosis factor (TNF), soluble TNF receptor (sTNFR), stem cell factor (SCF), protein carbonyl (PC), hepatocyte growth factor (HGF), collagen (Col), matrix metalloproteinase (MMP), basic fibroblast growth factor (bFGF).

An investigative study of CKD patients was conducted to identify novel biomarkers of progressive CKD and to validate emerging biomarkers. The aim was to develop a model for accurately predicting progressive CKD. A discovery-based approach using the CKD QLD Registry, termed the Biomarker Discovery cohort, identified 13 biomarkers that differed in baseline expression between CKD patients who subsequently progressed or who did not progress, in CKD. Several of these biomarkers, in addition to emerging biomarkers identified in the literature, were also screened in the CKD Biobank cohort, termed the Predictive Model cohort, where 10 biomarkers were found to differ between progressive and non-progressive CKD patients (Figure 3). Predictive models, termed the DROP CKD models, were developed based on biomarker panels representing the pathophysiological processes of progressive CKD, traditional kidney function measurements, and common CKD comorbidities. These predictive models were more accurate at predicting progressive CKD than current traditional kidney function measurements.

The DROP CKD models were created to predict future progression events based on the expression of a selected biomarker panel at baseline. Several predictive models for progressive CKD have been created. The most robust appears to be The Kidney Failure Risk Equation that was created in a cohort of >700,000 patients across the globe [9]. While promising, this model lacks in several areas. Patients with eGFR category G1 and G2 CKD and a definition of progressive kidney function decline of ≥30% decline in eGFR from baseline, were not included in the construction of The Kidney Failure Risk Equation. This is problematic because as G1 and G2 CKD patients are still at risk of experiencing a ‘progressive’ decline in kidney function, as shown in the research presented here, and published previously [15,16]. Additionally, the Kidney Failure Risk Equation did not include novel or emerging biomarkers in its development. The research presented here is some of the first showings that inclusion of novel and emerging biomarkers of progressive CKD, in addition to kidney function measurements and clinical information traditionally found in patient health records, improves prediction accuracy. Studies using the Scottish Diabetes Research Type 1 Bioresource and the Finish Diabetic Nephropathy cohorts showed that biomarker panels that included KIM-1 and CD27 greatly improved accuracy [15,17]. Moreover, a novel urinary biomarker panel, termed CKD273, and its sub-panels, were more accurate at predicting progressive CKD in the lower grade eGFR categories [16,18].

The tissue injury biomarkers osteopontin and TF were screened in the Predictive Model cohort. These biomarkers were increased in CKD patients classified as progressive. Osteopontin has received little attention as a biomarker of progressive CKD, but is known to be inversely correlated with eGFR [19,20]. Additionally, TF has not previously been associated with progressive CKD, but hypercoagulability is known to occur in CKD patients [21,22]. To our knowledge, this is the first study to associate increased osteopontin and TF levels with progressive CKD.

Despite being involved in a range of kidney diseases, mast cells have received little attention in the context of progressive CKD. SCF, a major regulator of mast cell activity, and the mast cell-specific protease tryptase, were screened in the Predictive Model cohort. This is the first study to record an association of SCF with progressive CKD, but considering increased mast cell activity has been observed in a range of kidney diseases [23,24,25,26,27,28,29,30,31], the SCF-progressive CKD association is perhaps unsurprising. Little research has been conducted into the association of tryptase with progressive CKD; however, the Renal Impairment in Secondary Care Study observed an association between elevated tryptase levels and progression towards kidney failure [32].

TNF-α and its soluble receptors sTNFR-I and sTNFR-II are major regulators of inflammation. While TNF-α was unchanged between progressive and non-progressive patients of the Predictive model cohort, sTNFR-I and sTNFR-II were increased in progressive patients of this cohort. Similar observations have been shown previously. The TNF-α observation is at odds with those in the Chronic Renal Insufficiency Cohort Study where plasma TNF-α was associated with a rapid reduction in kidney function [33]. In contrast nephropathy, increased sTNFR-I and sTNFR-II correlated with kidney function decline [34] and in type 1 and type 2 diabetes mellitus cohorts, they were associated with worse clinical outcomes [35,36,37,38]. Additionally, sTNFR-I was associated with an increased risk of progressive CKD in a community and CKD population [39,40].

Kidney measurements, such as sCr, urea, Alb, eGFR and ACR have been studied extensively in the context of CKD and its progression [4]. In both cohorts used here, patients classified as progressive demonstrated increased sCr and urea, while eGFR was decreased. The protein creatinine ratio was increased in progressive CKD patients of the Biomarker Discovery cohort. However, increased kidney damage was not observed in patients classified as progressive in the Predictive Model cohort, with Alb and ACR being unchanged between patient groups. Although increased Alb is a known biomarker of CKD progression and has been used in other clinical tools for predicting progressive CKD [9], Alb has been observed as being less accurate when predicting CKD progression in early eGFR categories than more advanced categories in addition to patients with advanced eGFR categories having normoalbuminuria [16,41]. With reduced kidney function in progressive CKD patients, a reduction in the ability of the kidney to filter excess electrolytes from the blood is expected. 

Haematocrit and haemoglobin, as biomarkers of anaemia, were reduced in progressive CKD patients of the Biomarker Discovery cohort. This observation in the Biomarker discovery cohort was not unexpected as anaemia is a known complication of CKD. The current results agree with previous research showing anaemia as an indicator for worse CKD outcomes [42]. The inverse association between kidney failure and haemoglobin levels was also observed in non-diabetic CKD and autosomal dominant polycystic kidney disease G2-G5 CKD patients [43,44]. Erythropoiesis-stimulating agents slowed progressive CKD in a non-dialysis dependent population [45].

Bicarbonate, a biomarker of metabolic acidosis, was reduced in both cohorts. Reduced bicarbonate has previously been associated with a higher risk of progressive CKD in several studies, including in children with glomerular disease, the AASK (African American Study of Kidney Disease and Hypertension) Study, and a CKD population sourced from a USA tertiary care centre [46,47,48]. The Modification of Diet in Renal Disease (MDRD) study showed that reduced serum bicarbonate levels were associated with the increased risked of kidney failure [49]. Chloride, another metabolic acidosis biomarker, was increased in progressive CKD patients, but only in the Biomarker Discovery cohort. Increased serum chloride was associated with lower baseline kidney function in the CKD-ROUTE (CKD Research of Outcomes in Treatment and Epidemiology) study [50]. In a G3-G4 CKD cohort, higher serum chloride levels were associated with worse kidney function decline, but not with a ≥30% decline in eGFR in a fully adjusted model [51].

As biomarkers of mineral and bone disease, measurements for phosphate and calcium were available in both cohorts, while parathyroid hormone was available only in the CKD QLD Registry. Previously, elevated phosphate levels have been associated with an increased risk of CKD progression and worse clinical outcomes in CKD cohorts [52,53], and is an autosomal dominant polycystic kidney disease cohort where increased phosphate levels were associated with kidney failure [46]. Calcium levels were decreased only in the progressive patients of the CKD QLD Registry cohort and were unchanged in the Predictive Model cohort. Furthermore, parathyroid hormone was elevated in progressive patients of the Biomarker Discovery cohort. According to our understanding, this is one of the first studies to investigate the association between calcium biomarkers and parathyroid hormone with progressive CKD. Considering that kidney function decline is associated with deterioration of mineral homeostasis and disruption to tissue and circulating levels of phosphate, calcium and parathyroid hormone [54,55], that the increase in calcium and parathyroid levels is expected in progressive CKD patients.

A composite outcome was used to define progressive CKD in both cohorts and was found to distinguish between patients who experienced progressive CKD and those who did not. This was based on a ≥30% decline in eGFR from baseline, initiation of dialysis, or receipt of a kidney transplant. In both cohorts, patients classified as progressive experienced a maximum eGFR percentage decline that was ~2-4 fold greater than those classified as non-progressive. Progressive patients of the Biomarker Discovery cohort demonstrated a steep, continuous decline, while non-progressive patients demonstrated an upward trajectory. Both patient groups of the Predictive Model cohort demonstrated downward trajectories; however, progressive patients demonstrated a steeper decline. The composite outcome used over-classified progression in the Biomarker Discovery cohort and under-classified progression in the Predictive Model cohort.

This investigation was limited in three aspects. Firstly, the DROP CKD models were constructed using a relatively small patient cohort. Secondly, several biomarkers that were associated with progression in the Biomarker Discovery cohort were not available from patient records in the Predictive Model cohort. Thirdly, the longer follow-up time of progressive patients in the Biomarker Discovery cohort and the more advanced eGFR category of progressive patients of the Predictive Model cohort conferred a slight risk of progressive CKD. Future studies accounting for these limitations are required to validate the screened biomarkers and biomarker panels.

The primary benefit of the approach of the DROP CKD models, and other predictive models [9,15,16], is its ability to be integrated into the public medical infrastructure. These biomarkers are measured in biospecimens, such as venous blood or urine, that are collected via minimally invasive procedures by a phlebotomist, a position that does not require highly specialised training. Furthermore, with the addition of the relevant assay kits, pathology laboratories have the infrastructure required to screen for these proteomic biomarkers. A major hurdle to deploying a predictive model of progressive CKD, such as these, would be in rural and remote regions of a country and in developing countries where public medical infrastructure is often insufficient to support it.

While the DROP CKD model shows promising results, future efforts are required to develop a clinically useful tool for predicting progressive CKD. Efforts are needed in identifying novel biomarkers of progressive CKD and using advanced statistical analysis in predictive model construction. Two high throughput approaches for identifying novel biomarkers are proteomics and metabolomics. Previously, proteomics has been utilised in a large-scale study where 273 urinary biomarkers that differed between healthy controls and CKD patients were identified. These findings were subsequently used in studies attempting to create predictive CKD models [16,18]. Metabolomic studies in CKD patients have focused on the blood and have been performed in small clinical cohorts [56,57,58]. Finally, as an advanced statistical approach, machine learning is being adopted in clinical CKD research. It has been used in several studies, including used with comorbidity data to predict kidney replacement therapy within 12 months of CKD diagnosis [59], and creation of biomarker panels using kidney measurements, dyslipidaemia biomarkers, serum sodium, and c-reactive protein to determine progressive CKD [60].

The research presented here has identified several novel biomarkers and validated several emerging biomarkers of progressive CKD. It has contributed to the growing body of literature that supports the use of novel and emerging biomarkers of CKD progression to improve the accuracy of models for predicting progressive CKD. It also supports the benefit of building predictive models from biomarkers that represent the pathophysiological processes of progressive CKD, traditional kidney measurements, and common CKD comorbidities. However, to create a successful clinical tool for predicting progressive CKD, more biomarker research is required, and more sophisticated approaches need to be used in its creation.

## Figures and Tables

**Table 1:** Patient Characteristics of the Biomarker Discovery and Predictive Model cohorts.

Characteristic	All	Progressive	Non-Progressive	*p*-Value
*Biomarker Discovery cohort*	418	183 (43.8)	235 (56.2)	
Age (Years)	67.2 ± 14.2	67.3 ± 14.2	67.1 ± 14.1	n.s.
Gender				
Male	210 (50.2)	99 (54.1)	111 (47.2)	n.s.
Female	208 (49.8)	84 (45.9)	124 (52.8)	
Body Mass Index ^a^	30.9 ± 7.8	30.9 ± 7.8	30.9 ± 7.9	n.s.
eGFR category				
G2	23 (5.5)	8 (4.4)	15 (6.4)	n.s.
G3a	77 (18.4)	30 (16.4	47 (20.0)	
G3b	171 (40.9)	71 (38.8)	171 (42.6)	
G4	147 (35.2)	74 (40.4)	147 (31.1)	
Follow-up (Years)	4.2 ± 1.6	4.6 ± 1.5	3.9 ± 1.6	****
*Predictive Model cohort*	62	33 (53.2)	29 (46.8)	
Age (Years)	53.3 ± 11.3	54.4 ± 10.4	52.1 ± 12.3	n.s.
Gender				
Male	37 (59.7)	23 (69.7)	14 (48.3)	n.s.
Female	25 (40.3)	10 (30.3)	15 (51.7)	
Body Mass Index ^b^	30.3 ± 6.4	30.3 ± 5.5	30.3 ± 7.4	n.s.
eGFR category				
G1	10 (21.0)	3 (9.1)	10 (34.5)	*
G2	11 (17.7)	6 (18.2)	5 (17.2)	
G3a	10 (16.1)	4 (12.1)	6 (20.7)	
G3b	13 (21.0)	8 (24.2)	5 (17.2)	
G4	15 (24.2)	12 (36.4)	3 (10.3)	
Follow-up (Years)	1.4 ± 0.5	1.3 ± 0.4	1.4 ± 0.4	n.s.

Categorial variables are presented as count (%), while nominal variables are presented as $\bar{x}\pm \mathrm{SD}$. ^a^ 407. ^b^ 57. Abbreviations: Estimated glomerular filtration rate (eGFR), non-significant (n.s.), *p* < 0.05 (*), *p* < 0.0001 (****).

**Table 2:** Biomarkers that differ between progressive and non-progressive CKD patients.

Biomarker (units)	Progressive	Non-Progressive	Pseudo-R^2^	*p*-Value
*Biomarker Discovery cohort*				
Bicarbonate ^a^ (mmol/L)	24.8 ± 3.3	25.8 ± 3.4	0.11	0.001
Calcium ^a^ (mmol/L)	2.31 ± 0.13	2.33 ± 0.11	0.11	0.01
Chloride ^a^ (mmol/L)	104.8 ± 4.1	103.8 ± 4.2	0.11	0.001
Ferritin ^a^ (µg/L)	210.6 ± 258.2	142.2 ± 125.5	0.10	0.05
Serum Creatinine ^a^ (µmol/L)	173.8 ± 68	153.5 ± 55.1	0.11	0.01
eGFR ^a^ (mL/min/1.73 m^2^)	34.6 ± 14.3	37.9 ± 14	0.09	0.05
Protein Creatinine Ratio ^b^ (mg/L)	93.2 ± 133.1	67.8 ± 106.4	0.16	0.01
Haematocrit ^c^	0.37 ± 0.05	0.39 ± 0.05	0.14	0.0001
Haemoglobin ^c^ (g/L)	120.4 ± 17.6	129.2 ± 16.8	0.14	0.0001
Parathyroid hormone ^a^ (ng/L)	131.3 ± 97.8	81.5 ± 50.9	0.25	0.01
Alkaline Phosphatase ^c^ (U/L)	90.9 ± 34	84.2 ± 29.3	0.13	0.05
Phosphate ^a^ (mmol/L)	1.2 ± 0.2	1.1 ± 0.2	0.10	0.01
Urea ^a^ (mmol/L)	14.6 ± 6.6	12.1 ± 5.7	0.10	0.01
*Predictive Model cohort*				
Bicarbonate ^d^ (mmol/L)	23.6 ± 4	25.9 ± 2.6	0.26	0.01
eGFR ^e^ (mL/min/1.73 m^2^)	46.3 ± 30.6	74.8 ± 39.9	0.11	0.01
Osteopontin ^d^ (ng/mL)	39.5 ± 24.8	28.8 ± 22.1	0.13	0.05
Stem cell factor ^d^ (pg/mL)	181.3 ± 79.7	113.6 ± 47.8	0.26	0.001
Serum Creatinine ^d^ (µmol/L)	223.3 ± 110.8	124.4 ± 63.4	0.26	0.001
Tissue factor ^d^ (pg/mL)	89.9 ± 34.7	66.9 ± 21.7	0.20	0.01
sTNFR-I ^e^ (ng/mL)	3.8 ± 1.6	2.7 ± 1.5	0.09	0.01
sTNFR-II ^d^ (ng/mL)	8.4 ± 3.2	6.8 ± 3.5	0.11	0.05
Tryptase ^d^ ng/mL)	5.9 ± 3.7	4.1 ± 1.9	0.19	0.01
Urea ^d^ (mmol/L)	14 ± 6.3	9.5 ± 5.6	0.15	0.01

Logistic regression model: ^a^ Biomarker + Dx + follow-up, ^b^ biomarker + age + Dx + follow-up, ^c^ biomarker + gender + Dx + follow-up, ^d^ biomarker + Dx, ^e^ biomarker. Abbreviation: Chronic kidney disease (CKD), estimated glomerular filtration rate (eGFR), soluble tumour necrosis factor receptor (sTNFR), kidney disease diagnosis (Dx).

## Supplementary Materials

The following are available online at https://www.mdpi.com/2227-9059/8/12/606/s1.
