# Supplementary material for: Development of a Biomarker Panel to Distinguish Risk of Progressive Chronic Kidney Disease
Source: Biomedicines. 2020 Dec 14;8(12):606. doi: 10.3390/biomedicines8120606 (PMC7764886; doi:10.3390/biomedicines8120606)
Supplement: Supplementary file 1 [file biomedicines-08-00606-s001.pdf]

biomedicines

MDPI

Table S1. Biomarkers screened in the Biomarker Discovery cohort

Biomarkers		
Alanine Aminotransferase	Gamma-GT	Protein Creatinine Ratio
Alanine Transaminase	Globulin	Total Protein
Albumin	Glucose	Red Cell Count
Alkaline Phosphatase	Haematocrit	Serum Creatinine
Total Alkaline Phosphatase	Haemoglobin	Sodium
Anion Gap	HDL Cholesterol	Transferrin
Aspartate Aminotransferase	HDL Cholesterol Ratio	Transferrin Saturation
Aspartate Transaminase	Iron	Transferring Iron Binding Cap
Basophils	Lactate Dehydrogenase	Triglycerides
Bicarbonate	LDL Cholesterol	Urate
Bilirubin	Lymphocytes	Urea
Total Bilirubin	Magnesium	Urea Creatinine Ratio
Calcium	Mean Cell Haemoglobin	Uric Acid
Calcium Corrected	Mean Corpuscular Volume	Urine Creatinine
Calculated Osmolality	Monocytes	Urine Creatinine, Random
Chloride	Neutrophils	Urine Protein, Random
Cholesterol	Other Anions	Total Urine Protein
C-Reactive Protein	Parathyroid hormone	VLDL Cholesterol
eGFR	Phosphate	White Cell Count
Eosinophils	Platelets	
Ferritin	Potassium	

Abbreviations: estimated glomerular filtration rate (eGFR), high-density lipoprotein (HDL), low-density lipoprotein (LDL), very low-density lipoprotein (VLDL).

Table S2A. Biomarker Discovery cohort: **p**rogression status and baseline biomarkers concentration

Biomarker (units)	Progressive	Non-progressive	Pseudo-R^2	p-value
Albumin [a] (g/L)	38.6 ± 5.1	39.5 ± 6.1	0.09	n.s.
Alanine Aminotransferase [a] (U/L)	21.8 ± 16.5	22.7 ± 16.2	0.07	n.s.
Alanine Transaminase [b] (U/L)	21.5 ± 12.7	21.8 ± 11	0.15	n.s.
Total Alkaline Phosphatase [a] (U/L)	93.1 ± 46.8	91.6 ± 48.5	0.08	n.s.
Anion Gap [b] (mmol/L)	8.5 ± 2.9	8.6 ± 3.1	0.11	n.s.
Aspartate Aminotransferase [a] (U/L)	25.2 ± 29.5	24.3 ± 11.6	0.08	n.s.
Aspartate Transaminase [b] (U/L)	22.8 ± 13.3	21.5 ± 6.1	0.15	n.s.
Basophils [a] (10^9/L)	0 ± 0.1	0 ± 0.1	0.08	n.s.
Bilirubin [c] (μmol/L)	7.2 ± 3.5	8 ± 3.4	0.01	n.s.
Total Bilirubin [a] (μmol/L)	9.6 ± 4.8	10.3 ± 5.1	0.09	n.s.
C-Reactive Protein [a] (mg/L)	12.8 ± 18.1	7.6 ± 5.8	0.14	n.s.
Calcium Corrected [a] (mmol/L)	2.4 ± 0.1	2.4 ± 0.1	0.08	n.s.
Cholesterol [a] (mmol/L)	4.2 ± 1.1	4.4 ± 1	0.08	n.s.
HDL Cholesterol [c] (mmol/L)	1.1 ± 0.4	1.2 ± 0.3	0.01	n.s.
HDL Cholesterol Ratio [c]	3.8 ± 1.2	3.9 ± 1.2	0.00	n.s.
VLDL Cholesterol [d] (mmol/L)	0.8 ± 0.4	0.9 ± 0.5	0.19	n.s.
LDL Cholesterol [c] (mmol/L)	2.0 ± 1.0	2.3 ± 0.9	0.02	n.s.
Eosinophils [a] (10^9/L)	0.4 ± 0.7	0.3 ± 0.7	0.08	n.s.
Gamma-GT [a] (U/L)	42.9 ± 52.5	41.2 ± 61	0.09	n.s.
Globulin [a] (g/L)	30.6 ± 5.4	29.7 ± 4.1	0.08	n.s.
Glucose [a] (mmol/L)	7.3 ± 3.4	6.4 ± 2.3	0.09	n.s.
Iron [a] (μmol/L)	13.1 ± 6.7	13.1 ± 3.7	0.09	n.s.
Lactate Dehydrogenase [b] (U/L)	216.7 ± 62.4	212.5 ± 58.8	0.08	n.s.
Lymphocytes [b] (10^9/L)	2.8 ± 4.5	2.7 ± 4	0.08	n.s.

Logistic regression utilised the variables: [a] biomarker + kidney disease diagnosis + follow-up time, [b] biomarker + gender + kidney disease diagnosis + follow-up time, [c] biomarker, [d] biomarker + age + gender + follow-up time Abbreviation: high-density lipoprotein (HDL), very low-density lipoprotein (VLDL), low-density lipoprotein (LDL), estimated glomerular filtration rate (eGFR).

Table S2B. Biomarker Discovery cohort: **p**rogression status and baseline biomarkers concentration

Biomarker (units)	Progressive	Non-progressive	Pseudo-R^2	p-value
Magnesium [a] (mmol/L)	0.8 ± 0.1	0.9 ± 0.1	0.03	n.s.
Mean Corpuscular Volume [b] (fL)	90.3 ± 6.3	90.7 ± 5.3	0.08	n.s.
Mean Cell Haemoglobin [c] (pg)	29.4 ± 2.5	29.9 ± 2	0.06	n.s.
Monocytes [b] (10^9/L)	0.9 ± 1.4	0.8 ± 1.1	0.08	n.s.
Neutrophils [b] (10^9/L)	6.1 ± 9.1	6.1 ± 9.5	0.08	n.s.
Calculated Osmolality [d] (mmol/kg)	296.5 ± 8.7	293.4 ± 8.7	0.14	n.s.
Other Anions [b] (mmol/L)	15.3 ± 2.1	15.1 ± 2.6	0.07	n.s.
Platelets [b] (10^9/L)	252.8 ± 77.7	245.8 ± 69	0.09	n.s.
Potassium [b] (mmol/L)	4.6 ± 0.5	4.6 ± 0.5	0.09	n.s.
Total Protein [b] (g/L)	69.4 ± 6.3	70.1 ± 4.8	0.10	n.s.
Red Cell Count [b] (10^{12}/L)	16.6 ± 159.9	4.4 ± 0.6	0.09	n.s.
Sodium [b] (mmol/L)	139 ± 3	139 ± 3	0.09	n.s.
Transferrin [b] (g/L)	2.2 ± 0.4	2.4 ± 0.4	0.24	n.s.
Transferrin Saturation [b]	24.0 ± 12.7	23.1 ± 6.5	0.10	n.s.
Transferring Iron Binding Cap [e] (μmol/L)	58.6 ± 10.2	57.7 ± 9.8	0.08	n.s.
Triglycerides [b] (mmol)	1.8 ± 0.9	1.9 ± 1.1	0.07	n.s.
Urate [d] (mmol/L)	0.5 ± 0.1	0.4 ± 0.1	0.15	n.s.
Urea Creatinine Ratio [d]	79.3 ± 23.3	74.9 ± 24.3	0.15	n.s.
Uric Acid [b] (mmol/L)	0.5 ± 0.1	0.5 ± 0.1	0.08	n.s.
Urine Creatinine [f] (mmol/L)	8.4 ± 4.8	8.6 ± 4.3	0.08	n.s.
Urine Creatinine, Random [a] (mmol/L)	9 ± 5.1	8.2 ± 4.8	0.00	n.s.
Urine Protein, Random [a] (mmol/L)	693.9 ± 986.4	536.6 ± 945.6	0.00	n.s.
Total Urine Protein [f] (mg/L)	731.7 ± 1383.2	362.4 ± 534.3	0.09	n.s.
White Cell Count [b] (10^9/L)	7.7 ± 2.3	7.8 ± 2.2	0.08	n.s.

Logistic regression utilised the variables: [a] biomarker, [b] biomarker + kidney disease diagnosis + follow-up time, [c] biomarker + kidney disease diagnosis, [d] biomarker + gender + kidney disease diagnosis + follow-up time, [e] biomarker + age + kidney disease diagnosis, [f] biomarker + kidney disease diagnosis.

Table S3A. Predictive Model cohort: **p**rogression status and baseline biomarkers concentration

Biomarker (units)	Progressive	Non-progressive	Pseudo-R^2	p-value
Albumin-Creatinine Ratio [a] (g/mol)	109.2 ± 175.1	106.1 ± 186.6	0.06	n.s.
Albuminuria [a] (mg/L)	1053.9 ± 2039	898.5 ± 1823.9	0.10	n.s.
Calcium [b] (mmol/L)	2.3 ± 0.1	2.3 ± 0.1	0.00	n.s.
CCL5 [a] (ng/mL)	7.1 ± 5.2	10.3 ± 12.6	0.11	n.s.
Chloride [b] (mmol/L)	105.7 ± 3.2	116.1 ± 59	0.01	n.s.
Chymase [c] (ng/mL)	3.2 ± 1.7	2.6 ± 1.3	0.08	n.s.
Collagen IV α1 [b] (pg/mL)	652.4 ± 281.9	592.7 ± 336.1	0.01	n.s.
Carboxypeptidase A3 [c] (ng/mL)	20.4 ± 3.8	20.1 ± 6	0.06	n.s.
Colony Stimulating Factor-2 [b] (pg/mL)	1.9 ± 5.5	0.8 ± 3.6	0.01	n.s.
Cystatin-C [a] (µg/mL)	1.4 ± 0.6	1.1 ± 0.6	0.12	n.s.
D-Dimer and FDPs [c] (µg/mL)	5 ± 2.7	5.3 ± 4.9	0.05	n.s.
Fetuin-B [b] (ng/mL)	3 ± 0.5	3.3 ± 0.8	0.04	n.s.
FGF-Basic [c] (pg/mL)	10.1 ± 12.7	9.3 ± 13.5	0.00	n.s.
Haematocrit [b]	0.4 ± 0	0.4 ± 0	0.03	n.s.
Haemoglobin [b] (g/L)	123.4 ± 14.3	130.6 ± 16.7	0.04	n.s.
Hepatocyte Growth Factor [b] (pg/mL)	136.7 ± 91.6	143 ± 102.2	0.00	n.s.
Interleukin-1β [a]	2.8 ± 6.3	0.6 ± 2	0.13	n.s.
Interleukin-6 [b] (pg/mL)	9.8 ± 42.3	2 ± 3.1	0.02	n.s.
Kidney Injury Molecule-1 [b] (pg/mL)	3.7 ± 15.5	1.1 ± 6	0.01	n.s.
MCP-1 [b] (pg/mL)	278.6 ± 258.5	273.8 ± 110.8	0.00	n.s.
MMP-1 [c] (ng/mL)	1.2 ± 1.1	1.7 ± 1.6	0.08	n.s.
MMP-9 [a] (µg/mL)	0.1 ± 0.1	0.1 ± 0.2	0.11	n.s.
NGAL [a] (ng/mL)	135.4 ± 61.4	112.1 ± 72.6	0.10	n.s.
Protein Carbonyls [c] (nmol/mg)	4.2 ± 1.5	5.8 ± 5.4	0.08	n.s.

Logistic regression utilised the variables: [a] biomarker + kidney disease diagnosis, [b] biomarker, [c] biomarker + gender. Abbreviations: chemokine ligand 5 (CCL5), fibrin degradation products (FDPs), fibroblast growth factor (FGF), monocyte chemoattractant protein-1 (MCP-1), matrix metalloproteinase (MMP), neutrophil gelatinase associate lipocalin (NGAL).

Table S3B. Predictive Model cohort: progression status and baseline biomarkers concentration

**Biomarker (units)**	**Progressive**	**Non-progressive**	**Pseudo-R^2**	**p-value**
Phosphate (mmol/L)	1.2 ± 0.3	1.1 ± 0.3	0.01	n.s.
TNF-α (pg/mL)	6.7 ± 3.0	6.5 ± 6.7	0.00	n.s.
Uromodulin (mmol/L)	4.1 ± 4.8	2.9 ± 2	0.02	n.s.

Logistic regression utilised the variable biomarker. Abbreviation: tumour necrosis factor (TNF).
